# Outbreak of Gastroenteritis in Tibetan Transit School, Dharamshala, Himachal Pradesh, India, 2006

**DOI:** 10.4103/0970-0218.51227

**Published:** 2009-04

**Authors:** Surender Nikhil Gupta, Naveen Gupta

**Affiliations:** Department of Health and Family Welfare, Regional Health and Family Welfare Training Centre (RHFWTC), Chheb, Kangra, Himachal Pradesh - 176 001, India

**Keywords:** Outbreak, gastroenteritis, Tibetan Transit School, Himachal Pradesh

## Abstract

**Background:**

On 28^th^ June, 2006, 55 cases of the gastroenteritis were reported among the hostellers of the Tibetan Transit School, Dharamshala. We investigated the outbreak to identify the source, propose control and preventive measures.

**Materials and Methods:**

We defined a case of the gastroenteritis as the occurrence of more than three smelly loose motions between 28^th^ June to 2^nd^ July, 2006 among some sections of the resident hostellers. We determined age and sex specific attack rate. We hypothesized it as a food borne beef meat outbreak. We conducted the case control study and collected the information about the food items consumed inside and outside the hostel at dinner using the standardized questionnaire. We calculated floor wise incidences of four hostels, odds ratios and attributable fractions. We interviewed food handlers. We lifted the seven rectal stool, four water and three samples from floor, kitchen and meat chopper room for culture and sensitivity.

**Results:**

116 cases patients of 802 hostellers met the case definition. The maximum attack rate (16%) was in the youngest group (15-20yrs) and nil in staff and 31-40 years age group with 5 overall attack rate as 14%. Sex specific attack rate was more (18%) in females. The floor wise incidences of the case patients were the highest in 2nd and 3rd floors, occupied by the youngest group. The median age was 20 yrs (Range 17-40 yrs). The most common symptoms were watery diarrhea (71/116, 61%) and bloody diarrhea-(45/116, 39%); abdominal pains-(87/116, 75%). Of the six food/water items examined, the food specific attack rate was highly statistically significant in the beef meat eaters (82% with PAF 71%), and Odds Ratio 19.19 (95% C.I. as 9.3-140). The food handlers & their cooking conditions in the kitchen were unhygienic. The food was not available for testing. Escherichia coli were detected in the samples from rectal stools, kitchen and meat chopper room. No fatality was reported.

**Conclusion/Recommendation:**

The beef meat purchased from outside was implicated for the explosive common source outbreak. The school authorities were counseled for hygienic food handling.

## Introduction

The World Health Organization (WHO) estimated that each year, nearly 2 billion episodes of diarrhea occur and that they lead to 4.6 million deaths among children under the age of five.(1) However, the incidences of diarrhea cases have reduced to 3 million due to the introduction of the Diarrheal Diseases Control Program in 1980-81.(2,3) In tropical regions, 15 to 40% of all deaths among children under 5 years old are related to diarrhea.(4) In India, diarrheal diseases are not only a major public health problem among children under the age of five,(5,6) but also in the lower belt of Himachal Pradesh, including, Hamirpur, Una, and especially Kangra where heavy rain falls are seen every year. Since diarrheal diseases are caused by 20 to 25 pathogens, vaccination, though an attractive disease prevention strategy, is not feasible. However, as the majority of childhood diarrheas are caused by V. cholerae, Shigellae dysenteriae Type 1, rotavirus, and Enterotoxigenic *Escherichia coli* (ETEC), which have high morbidity and mortality, vaccines against these organisms are essential for the control of epidemics.(7–9) Food-borne outbreaks caused by undercooked ground beef and drinking unpasteurized milk are common(10) in the Tibetan community but are seldom investigated. Severe disease and outbreaks of disease are most commonly due to serotype O157:H7, which, like most other highly pathogenic Shiga toxin Producing *E. coli* (STEC), colonize the large intestine by means of a characteristic attaching and effacing lesion.(11) The etiological role of STEC in causing individual infections as well as outbreaks in the developed countries has been identified,(12) while reports from developing countries like India and Bangladesh are sparse.(13)

The outbreak investigation of gastroenteritis in Tibetan Transit School (TTS) provided us with an excellent opportunity to investigate the different Tibetan cultural epidemiology vis a vis the Indian culture prevalent in the world-renowned hilly tourist place Dharamshala. The students who attend TTS are of various ages. The youngest student is 15 years old; the 764 students are within the ages of 15-30 years old. The 38 members of the staff are between 31 and 40 years old. After the acquisition of refugee status from Tibet to India these students were admitted into the school.(14) We investigated the outbreak with the following objectives: (1) confirming the existence of the outbreak, (2) identifying the source and mode of transmission, and (3) initiating control and preventive measures.

## Materials and Methods

On 29 June 2006, a casualty medical officer from a medical college reported a cluster of 55 cases of severe diarrhea to the district public health headquarters. This cluster had affected students from the Tibetan Transit School located in the village of Khaniara in the district of the Dharamshala. We lodged a Firsthand Information Report (FIR) to the district health authorities. However, this investigation was conducted in the context of a public health response to an outbreak and therefore an ethical committee review was not indicated. Informed consent was obtained from the case patients for this study. We entered and analyzed the data using a Microsoft Excel™ spreadsheet and Epics info, Version 3.3.2.

We defined a suspect hosteller case patient as the occurrence of three or more smelly loose motions in a 24-hour period, with either abdominal cramps or fever ≥ 100°F between 28^th^ June and 2^nd^ July 2006 among the students or the staff of the Tibetan Transit School. We defined the probable case patients as the case patients reporting with bloody diarrhea and the confirmed case patient as the suspected case patient in whom laboratory investigation confirms the presence of one or more food-borne pathogens in a clinical specimen.(15)

We searched the school for the hostellers' case patients on a room-to-room basis in the whole hostel and in the staff quarters. For this exercise, we constituted two teams of health workers. In each team, there were two health workers: one male and one female. Every case patient was interviewed with the semi-structured questionnaire in English and the Tibetan language (local school helper) for 20 minutes and also for case control study. The whole team was trained as well as supervised by two senior medical officers. We explained the purpose and processing of the samples. We randomly collected seven rectal swabs and three samples: one from the kitchen, one from the floor, and one from the meat chopper room for culture and sensitivities (C/S). We kept them in Cary Blair transport media to transport them to the department of microbiology of the local medical college, Kangra at Tanda. Antimicrobial susceptibility of the isolated pathogens was done using a disk diffusion technique. We could not lift any ingested food sample or their vomit from the case patients or from the spot, as there was lot of resistances, resentments, and refusals on this account.

For each hosteller case-patient, we collected information on demographic characteristics, environment, signs and symptoms, and possible risk factors. We produced a line listing of cases. We constructed an epidemic curve to describe the dynamics of the outbreak. We calculated the incidence by age and gender using denominators supplied by the management of the school. We mapped the distribution of the cases by the various rooms of the four hostels and calculated the floor wise incidences.

We interviewed the director and the staff of the school to obtain information about the school and the movements of the students. We interviewed food handlers about their method of preparation and their health status. We conducted an age and gender matched case control study. We defined our study population as those who stayed in the school during the last week of June and the first week of July 2006. We collected information regarding potential exposure to food and beverages in the days preceding the outbreak. We calculated the odds ratio and 95% confidence intervals using Epi-Info. Finally, we calculated the fraction of cases attributable to specific exposures using the classical formula, i.e., the proportion of cases exposed multiplied by the attributable fraction among exposed (Odds ratio-1/odds ratio).

We interviewed the water supply man, health inspectors, the medical officers from the local government primary health center, and nearby community members to enquire about the general water and sanitation situation. We visited the school, including the kitchen, the water sources, and the sanitation area. We collected four water samples from different areas of the school: the first sample was taken from outside the Irrigation and Public health, the second sample was taken from the kitchen IPH tap, the third sample was taken from the nearby *khud water,* and the fourth sample was taken from the stream flowing inside the school. The samples were taken to Dr. Rajinder Prasad Government Medical College (DRPGMC) Kangra at Tanda to test for contamination with coli form bacteria.

## Results

Our study results identified 116 hosteller case patients for a total 802 students and staff members (Overall Attack Rate (AR): 14%). In students, the AR was 15% (116/764) and in the staff members it was 0% (0/38, Table 1). There were no deaths. All cases patients were hospitalized. Of these, seven were referred for renal failure due to oliguria and one for Hemolytic Uremic Syndrome (HUS) to a tertiary reference center in Chandigarh, Union Territory. All the patients recovered. The incidence was highest among the younger students and higher among women. No staff members were affected. Hypothesis generating pointed to the fact that on 28 June 2006, some students had left the school to go in town and celebrate the birthday of their Guru, Karamappa. To celebrate, they ate beef in the morning and for lunch [Table 2]. The food handlers and their cooking conditions in the kitchen were unhygienic.

**Table 1:** Age and sex specific attack rate of gastroenteritis Tibetan Transit School, Khaniara, Himachal Pradesh, India, 2006

Characteristics	Total	Cases	Attack rate (%)
Age in years			
15-20	431	69	16
21-25	238	35	15
26-30	92	12	13
31-40	41	00	00
Students (15-30)	764	116	15
Staff (31-40)	38	00	00
Sex			
Male	573	75	14
Female	229	41	18
Total	802	116	14

**Table 2:** Frequency of selected exposures among gastro-enteritis cases and controls, Tibetan Transit School, Khaniara, Himachal Pradesh, India

	Frequency of exposure				Odds ratio	95% confidence interval
			
	Cases (n=116)		Controls (n=116)			
				
	#	%	#	%		
Wheat flour cakes	5	4	6	5	0.83	0.21-3.2
Cabbage	9	8	10	9	0.89	0.32-2.5
Noodles purchased outside	7	6	8	7	0.89	0.27-2.7
Beef purchased outside	89	77	17	15	19	9.3-140
Drinking school water supply	79	68	26	22	7.3	3.9-14
Drinking government water supply	65	56	26	22	4.4	2.4-8

**Figure 1:**
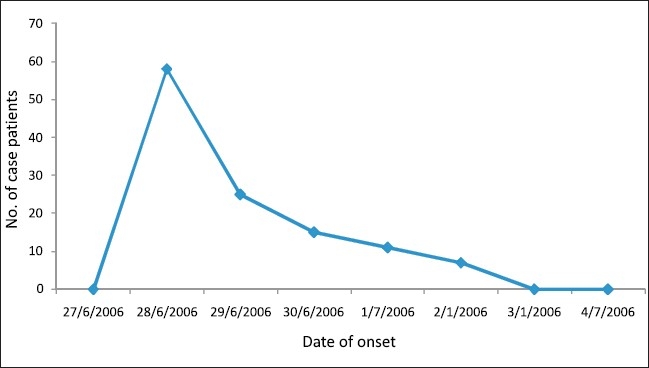
Cases of gastroenteritis by date of onset, Tibetan Transit School, Khaniara, Himachal Pradesh, India, 2006

In addition to watery diarrhea (71/116, 61%) and bloody diarrhea (45/116, 39%), case patients presented mainly with abdominal pains (87/116, 75%); [Table 3]. The first case had an onset of symptoms at 3.00 p.m. on 28 June 2006. On the same day, 57 other cases occurred, constituting the peak of the outbreak. There were subsequent cases the following days but the entire outbreak was over on 2 July 2006. No staff or community members were involved the outbreak [Figure 1].

**Table 3:** Frequency of symptoms among cases, Tibetan Transit School, Khaniara, Himachal Pradesh, India, 2006

Symptoms	Cases/Total	Percentage
Watery diarrhea	71/116	61
Bloody diarrhea	45/116	39
Abdominal pains	87/116	75
Vomiting	58/116	50
Nausea	52/116	45
Mass hysteria among the girls	45/116	39
Fever	12/116	10
Altered sensorium	12/116	10

### Incidence of the cases by the floors of the hostel

There are four resident hostels for the students of TTS: three are for the male students and one is for the female students. The floor-wise incidences of case patients for all the hostels are maximum in the 2^nd^ and 3^rd^ floors, respectively. Since the younger generation between the age group of 15-25 years old had occupied those floors and was mostly affected. The attack rates for the 2^nd^ and 3^rd^ floors ranged from 12.5% to 40.3% and 11.1% to 29.3%, respectively [Table 4].

**Table 4:** Incidences of the case patients in the hostels- Floor-wise-Tibetan Transit School, Khaniara, Himachal Pradesh, India, 2006

Floor no.	1^st^ boys hostel		2^nd^ boys hostel		3^rd^ boys hostel		4^th^ girls hostel	
				
	Cases/Total Attack rate (%)		Cases/Total Attack rate (%)		Cases/Total Attack rate (%)		Cases/Total Attack rate (%)	
1	0/61	0.0	16/70	23.0	5/64	7.8	0/76	0.0
2	8/64	12.5	0/66	0.0	25/62	40.3	19/78	24.35
3	7/63	11.1	10/58	17.3	4/65	6.2	22/75	29.3
Total	15/188	8.0	26/194	13.4	34/191	18.0	41/229	18.0

We recruited 116 cases and 116 controls. The median age of cases was 20 years (range: 15 to 30 years old) compared with 20 years old (range: 17 to 30 years old) for controls. The proportion of females was 37% among cases compared with 36% among control [Table 5]. Eating beef outside of the school was the risk factor most strongly associated with illness and that had the highest attributable fraction in the population (71%,). Other exposures associated with being a case included drinking water from the school water supply (attributable fraction in the population: 58%) and drinking water from the municipal water supply (attributable fraction in the population: 44%).

**Table 5:** Frequency matched by age group among gastroenteritis cases and controls, Tibetan Transit School, Khaniara, Himachal Pradesh, India, 2006

Age group (years)	Cases		Controls	
		
	Frequency	Percent	Frequency	Percent
15-20	69	59.5	67	57.8
21-25	34	29.3	33	28.4
26-30	13	11.2	16	13.8
31-40	00	00	00	00
Total	116	100.0	116	100.0

We collected a total of seven rectal swabs. Of these, five were collected after antibiotics. None of the samples were positive for Vibrio cholera. Rectal swabs grew positive for staphylococcus aureus (n=2), *Escherichia coli* (*E. coli*, n=5), and Klebseilla (n=5) while the C/S reports for the kitchen room, meat chopper room, and floor samples were *E. coli* for the former two while Enterococcus sp. and Klebseilla were for the floor specimen, respectively.

Many Tibetan private street vendors sell all kinds of food items in the vicinity of the school under unhygienic conditions. Beef consumption is not culturally acceptable in that largely Hindu area. However, Tibetan people have the practice of eating beef and other animals slaughtered months earlier and kept in the absence of cold chain because of the cold climate. It was unclear whether the students bought it from outside vendors or prepared it themselves.

The school had three sources of water supply: two sources of piped water (one private and one from the government) and a stream. The private piped water from the school came from surface waters collected higher in the mountain and two grounds dug water pumps in the campus as shown on the map. Upon inspection, the water tank was smelly and muddy, suggesting poor maintenance. There was no practice of chlorination. The government source of piped water came from an ill maintained and poorly chlorinated reservoir made in the mountains. The water from the stream was used for scrubbing the kitchen utensils. The laboratory of the medical college considered water samples from these three water sources unsatisfactory [Table 6].

**Table 6:** McCredie's tables for examination of water, milk, food, and air

Most probale number (mpn)/100 coliforms	Quality of water
Zero/100 ml of water	Excellent
1-3/100 ml of water	Good
4-9/100 ml of water	Suspicious
10 or >10/100 ml of water	Unsatisfactory
Presence of single *Escherichia coli*	Unsatisfactory

## Discussion

This gastroenteritis (GE) outbreak affected a large proportion of the students of TTS. A precise microbiological diagnosis was not possible. The distribution of cases over time suggested a common source outbreak. However, we identified three different risk factors for the illness: (1) eating beef outside of the school for a birthday celebration, (2) drinking water from the school water supply, and (3) drinking water from the municipal water supply.

Our bacteriological investigations led to the identification of three pathogens. *E. coli* was a generic isolation in the absence of characterization of the strain involved (EPEC, ETEC serotype O157 H7).(16) Usually, O157 H7 strains of *E. coli* are strongly associated with beef associated GE outbreaks but further testing could not be carried out at the local medical college microbiology laboratory because the frozen stools, sweeps, or isolates were not stored and the facilities were not available. Consumption of beef for the maximum number (86/116, 77%) of the case patients was the exposure most strongly associated with the illness and that accounted for the highest proportion of cases [Table 2]. Klebsiella is not a recognized cause of gastroenteritis. Staphylococcus is improbable because of the shorter incubation period.(17)

In our study, the absence of cases in the community suggests that they might have prepared the beef themselves and that it was not bought from the street. The C/S reports of *E. coli* in the kitchen and the meat chopper room indicated the cooking of beef there(18) facilitating the contamination of the beef.(19) The results of our case control study suggested that drinking water from the two water sources that supplied the school may also have been associated with the illness. Although the association was statistically significant, the strength of the association and the proportions of cases exposed were lower than for the beef. These two associations might have been causal or artifactual. However, a number of elements go against that hypothesis. First, the distribution of cases over time suggests a single source. Second, if the school water supply had been the source of infection, there would have been cases among staff members. Third, if the municipal water supply had been the source of infection, there would have been cases in the population.

## Conclusions

The most probable source of infection was the consumption of beef during the guru's birthday celebration.

This outbreak of GE effected a substantial proportion of resident students but could not be diagnosed conclusively (O157 H7 strain of *E. coli* could not be serotyped) using microbiological methods.

## Recommendations

On the basis of our findings, we can propose a number of recommendations.

Methods should be identified (i) to assess the circumstances of beef consumption in the area that may lead to outbreaks and (ii) preventive practices. This may include the identification of trading routes, dealers and all stakeholders, slaughtering practices, storage methods, and cooking recipes.

Educate the students as well as the cooks about safe food handling practices.
